# Podcast advertising by online sports betting platforms and alcohol brands, 2024-2025

**DOI:** 10.1093/haschl/qxag213

**Published:** 2026-08-19

**Authors:** Mark K Meiselbach, Soren Clarkwest, Benjamin Westermeyer, Matthew D Eisenberg

**Affiliations:** Department of Health Policy and Management, Johns Hopkins Bloomberg School of Public Health, Baltimore, MD 21205, United States; Johns Hopkins Center for Mental Health and Addiction Policy, Baltimore, MD 21205, United States; Department of Health Policy and Management, Johns Hopkins Bloomberg School of Public Health, Baltimore, MD 21205, United States; Johns Hopkins Center for Mental Health and Addiction Policy, Baltimore, MD 21205, United States; Department of Health Policy and Management, Johns Hopkins Bloomberg School of Public Health, Baltimore, MD 21205, United States; Johns Hopkins Center for Mental Health and Addiction Policy, Baltimore, MD 21205, United States; Department of Health Policy and Management, Johns Hopkins Bloomberg School of Public Health, Baltimore, MD 21205, United States; Johns Hopkins Center for Mental Health and Addiction Policy, Baltimore, MD 21205, United States; Department of Mental Health, Johns Hopkins Bloomberg School of Public Health, Baltimore, MD 21205, United States

**Keywords:** sports betting, gambling, advertising, podcasts, alcohol, marketing regulation, public health

## Abstract

**Introduction:**

Online sports betting has expanded rapidly in the United States following the 2018 Supreme Court decision in Murphy v. National Collegiate Athletic Association, with a growing body of research linking this expansion to increases in problem gambling, financial distress, bankruptcy filings, and intimate partner violence. Sportsbooks have invested heavily in advertising to acquire and engage customers. Podcasts may be a particularly attractive channel for sportsbook marketing, but little is known about the scale or targeting of this advertising.

**Methods:**

Using podcast advertising data from Podscribe for January 2024 through December 2025, we describe the podcast advertising estimated spending and volume for major online sportsbooks and compare them to the largest alcohol advertisers on podcasts.

**Results:**

In 2025, sports betting advertisers spent an estimated $63.4 million on podcast advertising, 2.9 times the $21.9 million spent by alcohol advertisers. DraftKings was estimated to have outspent all alcohol advertisers combined in 2025. DraftKings and FanDuel together accounted for 82% of estimated sports betting podcast spending, and The Joe Rogan Experience captured 13.9% of estimated sports betting podcast spending in 2025.

**Conclusion:**

The scale, format, and concentration of sportsbook podcast advertising merit attention from policymakers considering federal marketing regulations.

Key Points

- In 2025, sports betting advertisers spent an estimated $63.4 million on podcast advertising, 2.9 times the estimated $21.9 million spent by alcohol advertisers.
- DraftKings and FanDuel together accounted for 82% of estimated sports betting podcast spending, and The Joe Rogan Experience captured 13.9% of estimated sports betting podcast spending in 2025.
- Sports betting advertisers relied more heavily on host-read endorsements than alcohol advertisers.

## Introduction

The 2018 US Supreme Court decision in Murphy v. National Collegiate Athletic Association struck down the Professional and Amateur Sports Protection Act and opened the door for states to legalize sports betting.^1^ By the end of 2025, 39 states and the District of Columbia permitted some form of sports wagering, and Americans legally wagered a record $166.94 billion that year.^2^ The rapid expansion of online sports betting is a major public health concern, as its uptake could increase the prevalence of gambling addiction and related mental and behavioral health harms.^3,4^

Gambling advertising exposure has been linked to both gambling uptake and problem gambling.^5^ Yet most research, and most proposed regulation, has focused on broadcast television and social media channels, leaving open the question of how sportsbooks are reaching consumers through other media. Podcasts may be a particularly attractive channel for sportsbook marketing given that many of the most popular podcasts are sports-focused, but little is known about the scale or targeting of this advertising. Podcasts are a substantial and growing advertising market, with US podcast advertising revenue reaching an estimated $2.9 billion in 2025, a more than 50% increase since 2022.^6^

In this paper, we compare sportsbook podcast advertising to advertising by major alcohol brands, as alcohol is the closest legal analog: an adult-targeted product with established links between advertising and harms^7^ and self-regulation of advertising content as an industry,^8^ but no federal statute governing its marketing.

## Methods

We used advertising data from Podscribe, a podcast measurement platform that is used by major performance advertising agencies and companies to run podcast advertising campaigns. Podscribe continuously takes in podcast audio from thousands of shows across major platforms (iHeartMedia, SiriusXM, Wondery, NPR, Acast, Spotify) and applies audio- and transcript-based methods to detect each ad placement and record the advertiser, show, episode, and within-episode placement.^9^ While there are no quantitative estimates of Podscribe's comprehensiveness in the academic literature, the aggregate estimated expenditures for the top 1500 advertisers captured on Podscribe's platform in the 12 months preceding July 2026 summed to $2.87 billion (Appendix 1), within close range of the $2.9 billion estimated podcast advertising revenue in 2025 from external sources.^6^

The primary data used for this analysis were from January 2024 through December 2025 and focused on US markets. The most granular unit is a unique advertiser–episode combination (one row per distinct advertiser appearing on an episode), which we aggregated to the advertiser, show, industry, and month levels. From these data, we calculate each advertiser's estimated spend, unique show episodes carrying its ads, and downloads of those episodes. Estimated spend is modeled by Podscribe as estimated ad impressions multiplied by their proprietary benchmark cost-per-impression rates, with actual campaign pricing used where advertisers provide it. Each ad was classified as host-read (ie, the host of the podcast directly reading out the ad copy) or a produced commercial. For host-read ads, Podscribe distinguishes paid placements from organic brand mentions by identifying discrete ad segments within episode transcripts, using creative markers characteristic of advertisements (eg, sponsorship framing, promotional offers and codes, disclaimers) combined with human review. Produced commercials are more readily identified because they are prerecorded spots whose audio is distinct from the host's speech and reused across episodes. Additionally, the Podscribe platform includes demographic information for each podcast, which it sources from TransUnion via household IP address, as well as from social media among show followers on those platforms (eg, Facebook, X/Twitter).

While the Podscribe data cover advertisements across all industries, we focus on advertisers in 2 industries: online sportsbooks (DraftKings, FanDuel, BetMGM, and a combined category of all other sportsbooks) and alcohol advertisers (The Boston Beer Company, Molson Coors, Anheuser-Busch, and a combined category of all other alcohol advertisers). We first selected advertisers with a “Sports Gambling” or “Alcohol” industry designation in Podscribe's industry ranker table, which included 77 sportsbook advertisers and 106 alcohol advertisers. We then manually reviewed all selected advertisers and filtered out miscategorized ones (eg, 1-800-Gambler). We also consolidated advertisers with differing spelling or joint ownership (eg, “Truly”, “Twisted Tea”, and “Angry Orchard” grouped as “The Boston Beer Company”). Exclusions and consolidation decisions were made manually and then independently assessed by a second reviewer. The raw names, consolidated advertiser groupings, and inclusion/exclusion decisions are displayed in Appendices 2 and 3.

After these manual cleaning steps, our final data include 36 unique sportsbook advertisers and 53 unique alcohol advertisers. All data were accessed through Podscribe's public API, retrieving estimated spend by show and day for each identified advertiser.

We describe (1) advertiser totals and shares of estimated industry spending in 2025; (2) total estimated podcast advertising spending by industry and month; (3) episode downloads by industry and month; (4) the share of estimated spending across individual shows; and (5) the share of advertising delivered as host-read endorsements vs produced commercials. We assessed longitudinal estimated spend and episode downloads monthly from January 2024 to December 2025 to track seasonal trends across years. For all other analyses, we focus only on advertisements in 2025.

## Results

**Figure 1.**
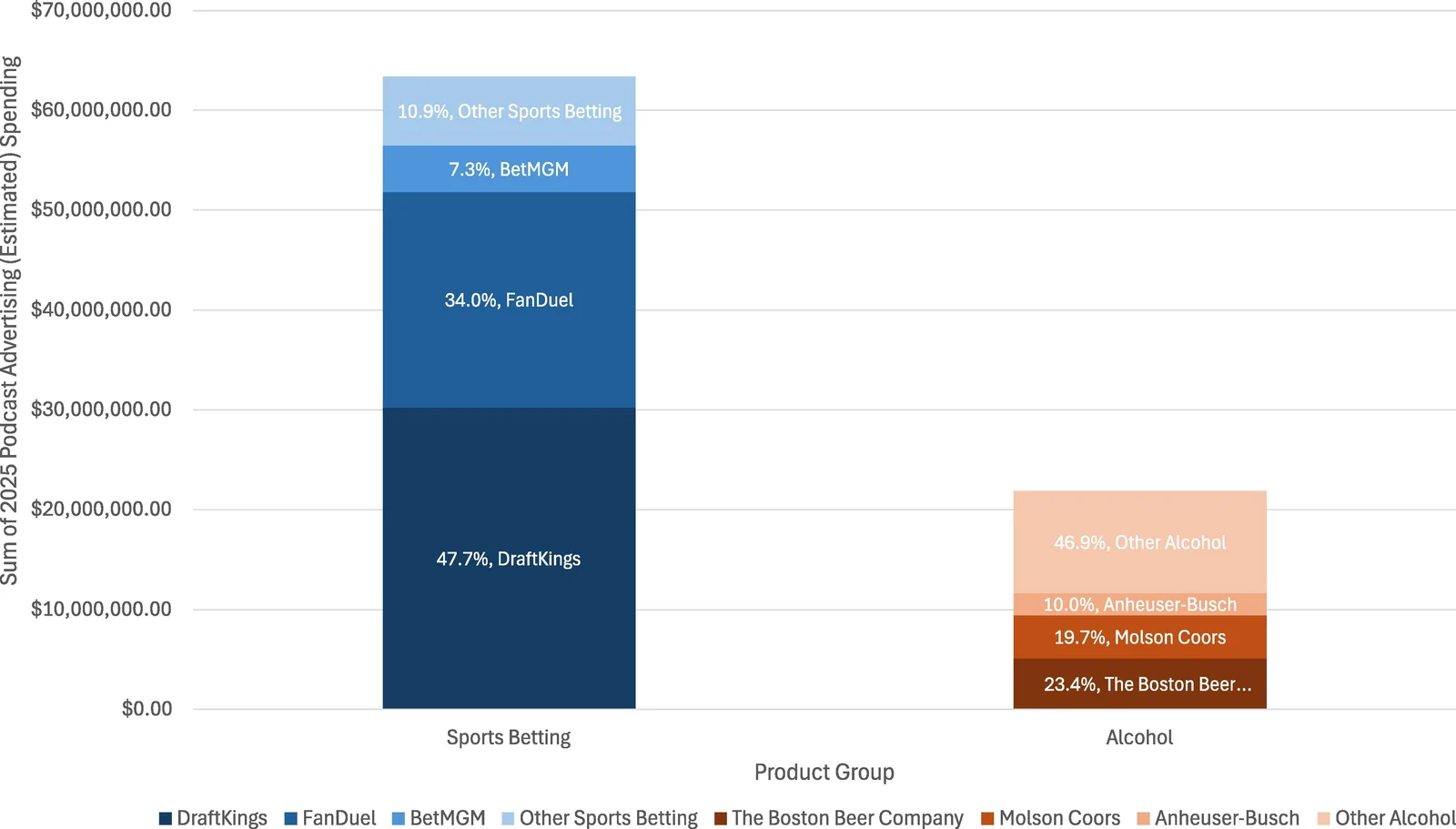
Total estimated podcast advertising spending by advertiser in sports betting and alcohol industries, 2025. Source: Authors’ analysis of Podscribe advertising occurrence data, 2025. Notes: Shares of estimated 2025 podcast advertising spending were calculated within each industry. Sports betting includes DraftKings, FanDuel, BetMGM, and a combined category of all other sportsbooks; alcohol includes The Boston Beer Company, Molson Coors, Anheuser-Busch, and a combined category of all other alcohol advertisers.

Online sportsbooks spent an estimated $63.4 million on podcast advertising in 2025, 2.9 times the estimated $21.9 million spent by alcohol advertisers on podcasts (Figure 1). DraftKings ($30.3 million) alone was estimated to have outspent all alcohol advertisers combined. Compared to alcohol advertising, sports betting advertising was more concentrated, with the 2 largest sports betting advertisers, DraftKings and FanDuel, comprising 81.7% of total estimated sports betting podcast advertising spend over this period, whereas The Boston Beer Company and Molson Coors, the 2 largest alcohol advertisers, comprised 43.1% of estimated total alcohol podcast advertising.

**Figure 2.**
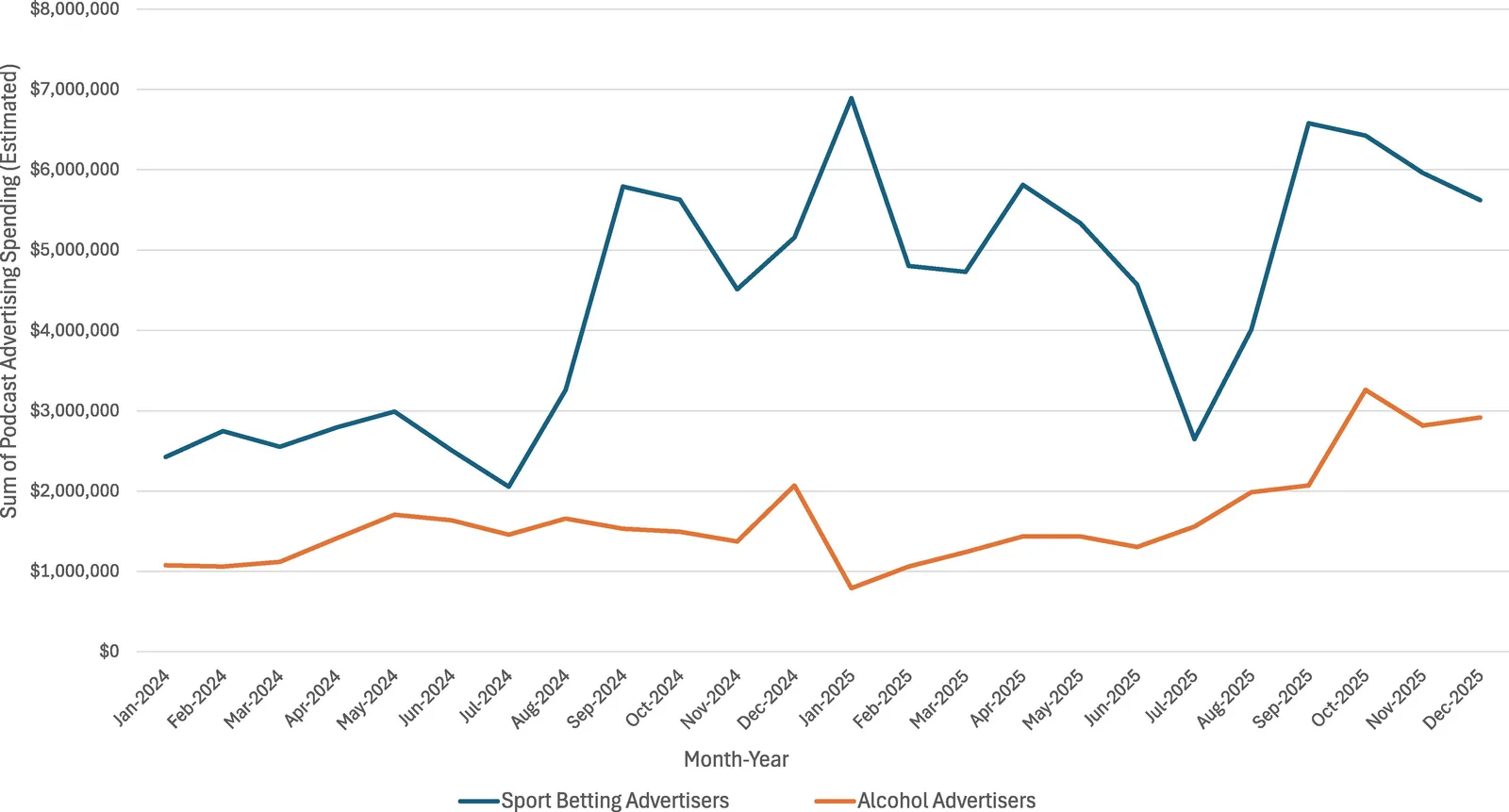
Monthly estimated podcast advertising spending by online sportsbooks and alcohol advertisers, January 2024–December 2025. Source: Authors’ analysis of Podscribe advertising occurrence data, January 2024–December 2025. Notes: Monthly estimated podcast advertising spending in US dollars was calculated for online sportsbook advertisers and alcohol advertisers on podcasts.

Sports betting podcast estimated spending rose 49% between calendar years 2024 and 2025 (from $42.4 million to $63.4 million), while alcohol estimated podcast spending rose 24% (from $17.6 million to $21.9 million) (Figure 2). Monthly sports betting estimated spending was seasonal, spiking at the beginning of the NFL season in September and October and again during the NFL playoffs in January. Alcohol estimated spending showed less seasonal variation but rose in the final quarter of 2025.

Total podcast downloads carrying a sports betting advertisement peaked at 387.8 million downloads in April 2025 (Appendix 4). Sports betting advertisers had advertisements on 2 to 10 times as many downloads in most months. However, the total number of downloads was similar in December 2025 across the 2 industries, even as sports betting advertisers spent nearly twice as much ($5.6 million vs $2.9 million).

Podcast advertising for both industries was heavily concentrated among a small number of shows, with substantial overlap across industries (Table 1). The top 20 shows carrying sports betting advertising accounted for 49.8% of estimated sports betting podcast spending in 2025, and the top 20 shows carrying alcohol advertising accounted for 63.8% of estimated alcohol podcast spending. The Joe Rogan Experience alone accounted for 13.9% of estimated sports betting podcast spending ($8.8 million) and 7.6% of estimated alcohol podcast spending ($1.7 million). Pardon My Take accounted for 8.1% of estimated sports betting spending and 17.2% of estimated alcohol spending. Five of the top 10 sports betting shows and 8 of the top 20 shows for each industry appeared on both industries’ lists. Overall, both sports betting and alcohol advertisers focused on sports podcasts (Appendix 5). Sports betting advertisers were estimated to spend $37.7 million on sports podcasts, compared to $13.2 million among alcohol advertisers. Additionally, audience demographics were similar between the 2 industries (Appendix 6). Audiences of both industries’ ads were overwhelmingly male (79.4% for sports betting and 78.6% for alcohol) and predominantly White (88.9% and 88.6%).

**Table 1.** Top 20 podcast shows carrying sports betting and alcohol advertising, by share of estimated industry spending, 2025.

Sports betting advertisers	Estimated spending total	Episodes	Total downloads	Proportion of estimated spending
The Joe Rogan Experience^a^	$8 830 038	225	805 614 525	13.9%
Pardon My Take^a^	$5 125 132	152	103 422 320	8.1%
The MeidasTouch Podcast	$2 070 674	760	633 594 520	3.3%
New Heights with Jason & Travis Kelce	$1 997 283	61	38 516 315	3.2%
The Bill Simmons Podcast^a^	$1 359 510	149	36 438 099	2.1%
The Dan Le Batard Show with Stugotz^a^	$1 337 214	958	53 876 004	2.1%
The Shawn Ryan Show^a^	$1 328 455	44	89 918 708	2.1%
Locked On NFL - Daily Podcast On The National Football League	$1 236 566	338	19 686 472	2.0%
Spittin Chiclets	$1 073 590	49	16 545 536	1.7%
The Ringer NBA Show	$969 826	138	4 769 832	1.5%
OverDrive	$761 586	622	22 937 494	1.2%
Stugotz and Company	$752 278	221	11 108 123	1.2%
The Bobby Bones Show	$726 955	349	13 963 141	1.1%
Call Her Daddy^a^	$712 651	47	50 489 280	1.1%
Fly on the Wall with Dana Carvey and David Spade	$623 561	102	14 610 582	1.0%
Club Shay Shay^a^	$580 774	487	353 547 877	0.9%
Conan O’Brien Needs A Friend^a^	$540 016	24	12 094 248	0.9%
This Past Weekend w/Theo Von	$523 995	32	44 018 688	0.8%
The Ryen Russillo Podcast	$518 948	69	7 559 226	0.8%
Fantasy Football Today	$509 186	233	13 077 591	0.8%
Other	$31 817 583	74 494	2 195 703 918	50.2%
**Alcohol advertisers**				
Pardon My Take^a^	$3 767 105	128	87 092 480	17.2%
The Dan Le Batard Show with Stugotz^a^	$3 627 328	1570	88 293 660	16.6%
The Joe Rogan Experience^a^	$1 654 326	20	71 610 180	7.6%
The Bill Simmons Podcast^a^	$1 237 119	113	27 634 263	5.7%
Conan O’Brien Needs A Friend^a^	$930 999	41	20 661 007	4.3%
Chicks in the Office	$378 877	72	7 891 920	1.7%
Unnecessary Roughness	$244 188	63	4 125 870	1.1%
Stuff You Should Know	$226 520	14	6 288 408	1.0%
The Shawn Ryan Show^a^	$221 934	5	10 218 035	1.0%
Club Shay Shay^a^	$212 162	212	153 905 852	1.0%
Throwbacks with Matt Leinart & Jerry Ferrara	$199 220	14	1 673 714	0.9%
KILL TONY	$168 158	9	26 888 940	0.8%
Call Her Daddy^a^	$151 636	4	4 296 960	0.7%
Giggly Squad	$148 304	7	3 172 946	0.7%
And That's Why We Drink	$138 788	10	989 430	0.6%
FantasyPros - Fantasy Football Podcast	$138 135	139	4 707 652	0.6%
Club 520 Podcast	$136 371	93	17 646 378	0.6%
Today, Explained	$129 284	13	3 177 863	0.6%
You Should Know Podcast	$128 410	10	3 572 320	0.6%
We’re Here to Help	$128 216	22	1 632 004	0.6%
Other	$7 910 356	13 258	798 842 460	36.2%

^a^Denotesshows that appear on both lists

Source: Authors’ analysis of Podscribe advertising occurrence data, 2025.

The 20 shows with the highest share of estimated industry podcast advertising spending were identified separately for sports betting and alcohol in 2025. Shares are expressed as a percentage of estimated total 2025 industry spending. Shows appearing on both industries’ top 20 lists are flagged.

**Figure 3.**
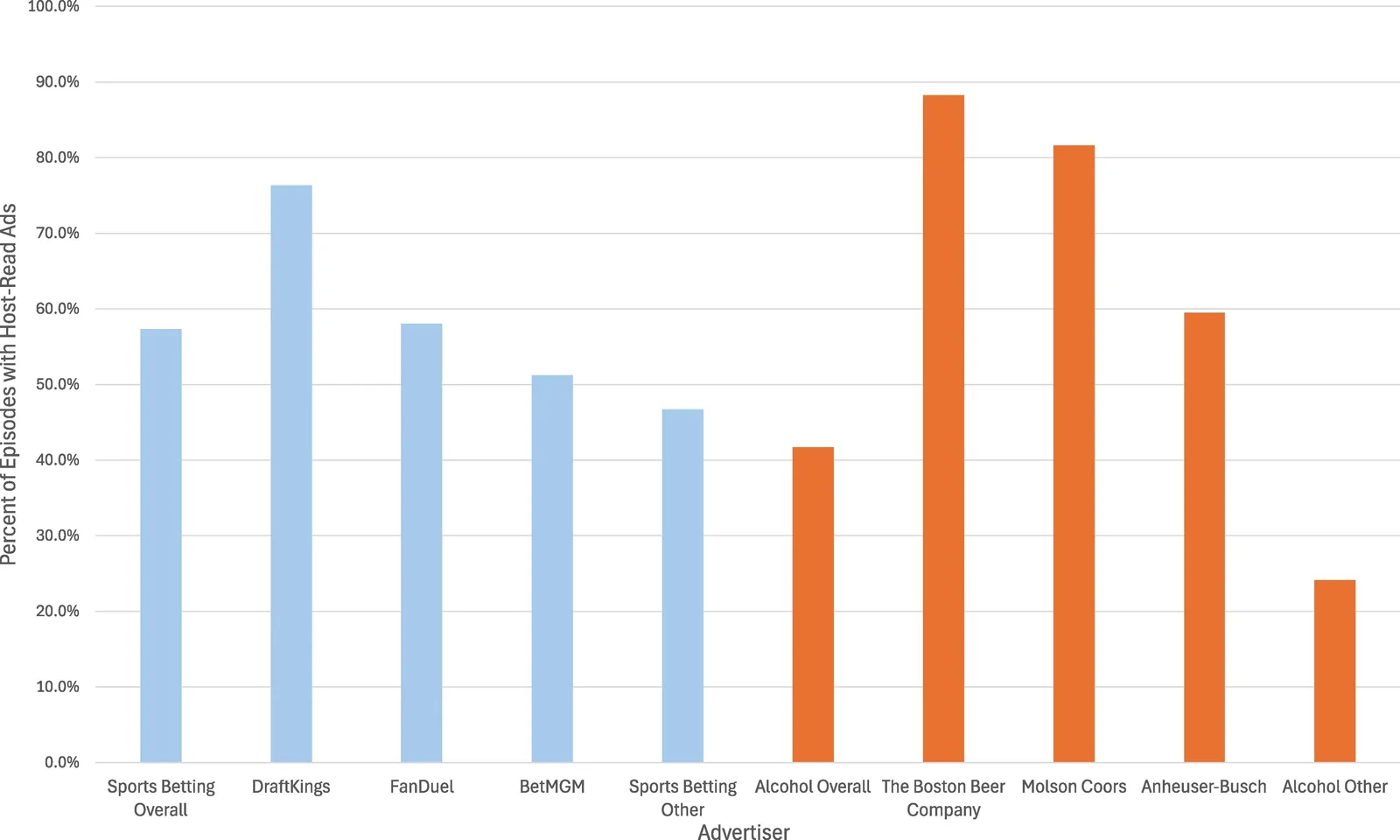
Share of podcast advertising episodes delivered as host-read endorsements, by advertiser, 2025. Source: Authors’ analysis of Podscribe advertising occurrence data, 2025. Notes: The share of advertising episodes classified by Podscribe as host-read was calculated for each advertiser in 2025. Host-read endorsements are delivered by the podcast host during the program.

Sports betting advertisers relied more heavily on host-read endorsements than alcohol advertisers (Figure 3). Across all sports betting podcast advertising episodes, 57.3% were host-read, compared to 41.7% among alcohol advertising episodes. Among major sportsbooks, DraftKings had the highest host-read share (76.3%). Among major alcohol advertisers, The Boston Beer Company and Molson Coors delivered the majority of their ads as host-reads (88.2% and 81.6%, respectively). However, smaller advertisers in both industries used host-read ads less frequently than the major advertisers (46.7% among other sports betting advertisers and 24.1% among other alcohol advertisers).

## Discussion

Our analysis offers several new descriptive facts about sportsbook advertising on podcasts. First, podcast advertising by online sportsbooks is substantial: sportsbooks spent an estimated $63.4 million in 2025 on podcast advertisements, 2.9 times what alcohol advertisers spent, and roughly 2% of the estimated $2.9 billion spent on podcast advertisements in 2025.^6^ Second, sportsbook podcast advertising is concentrated among 2 firms (DraftKings and FanDuel) and a small number of shows, notably The Joe Rogan Experience and Pardon My Take. Third, sportsbook advertising relies more heavily on host-read endorsements than alcohol advertising, a format that incorporates the advertisement more smoothly into the podcast content and may be more persuasive than produced commercials.^10^

These findings have implications for ongoing policy debates about sportsbook marketing. The United States regulates the advertising of adult-risk products inconsistently: cigarette advertising was banned from broadcast television and radio under the 1970 Public Health Cigarette Smoking Act, while alcohol advertising operates under voluntary industry codes that set demographic placement standards and content restrictions. Even as federal authority over tobacco marketing expanded through the 2009 Family Smoking Prevention and Tobacco Control Act and the Food and Drug Administration's 2016 Deeming Rule extending oversight to e-cigarettes, newer media created regulatory gaps, exemplified by the rapid growth of youth-targeted e-cigarette promotion on social media.^11-13^ Sports betting advertising on podcasts may present an analogous challenge where host-read endorsements closely resemble the social media marketing that eluded tobacco regulation.

Sportsbook advertising is subject to no federal broadcast restrictions comparable to those governing tobacco. Voluntary industry standards do exist, such as the American Gaming Association's Responsible Marketing Code for Sports Wagering and the operator-led Coalition for Responsible Sports Betting Advertising that set age-related placement standards and require responsible-gambling messaging.^14,15^ However, these codes are self-enforced without independent monitoring or penalties, and they do not restrict the volume of advertising or address podcast-specific formats such as host-read endorsements. Federal legislation under consideration, including the SAFE Bet Act, would restrict the timing of broadcast advertisements and certain promotional offers, such as bonus bets marketed as risk-free wagers (eg, “no sweat bets”) that in fact require additional betting,^16^ but would leave audio and podcast advertising largely untouched. This is a consequential omission as podcasts are a major channel for sportsbook marketing (DraftKings alone was estimated to have outspent all alcohol advertisers combined in 2025) and sportsbook marketing spend is concentrated on a small number of programs. Further, the prevalence of host-read endorsements in sportsbook podcast advertising is particularly relevant because these ads are often integrated into editorial content and may not be perceived as advertising by listeners, and there are currently no sponsorship-identification rules like those that exist in broadcast advertising (47 U.S.C § 317).^17^

Existing research on sports betting advertising has documented dose-response relationships between advertising exposure and gambling behavior, with stronger effects among higher-risk gamblers.^18^ That evidence base is built almost entirely on television and social media advertising. Features of the podcast environment may amplify these effects: host-read endorsements lend the credibility of a familiar voice, and listeners are typically attentive and alone. Further research is warranted to understand how podcast advertising specifically affects sports betting uptake, frequency, and problem gambling and whether host-read endorsements have effects distinct from those of produced commercials.

This analysis has limitations. First, Podscribe's data may not capture every podcast or platform; coverage may underrepresent independent shows or those distributed exclusively on video platforms, and episode download counts do not reflect video consumption of simulcast episodes. However, aggregate expenditures captured by Podscribe approximate external estimates of total US podcast advertising revenue. Second, spend estimates are modeled from proprietary cost-per-impression benchmarks and impression counts rather than observed transaction prices and may not reflect negotiated rates or in-kind arrangements between advertisers and publishers. Negotiated rates could diverge from benchmarks differentially between sportsbook and alcohol advertisers. Third, ad identification relies on Podscribe's automated detection and classification, which may contain errors. In particular, host-read ads could be confused with organic brand mentions, though paid reads are identified by creative markers (eg, sponsorship framing, promotional offers, required disclaimers) that are typically not included in editorial content. Fourth, we use downloads to estimate podcast show reach. However, some listeners may never listen to downloaded episodes, and some streaming audio listeners may not generate downloads.

## Conclusion

Online sportsbooks have become major podcast advertisers, outspending alcohol brands nearly 3-fold while relying heavily on host-read endorsements. This advertising is currently subject to no federal broadcast restrictions, and it is largely overlooked by leading reform proposals such as the SAFE Bet Act. As policymakers weigh how to address the public health consequences of sports betting, the scale, concentration, and format of podcast advertising warrant closer attention.

## Supplementary Material

qxag213_Supplementary_Data
