# Supplementary material for: Podcast advertising by online sports betting platforms and alcohol brands, 2024-2025
Source: Health Aff Sch. 2026 Aug 19;4(9):qxag213. doi: 10.1093/haschl/qxag213 (PMC13531462; doi:10.1093/haschl/qxag213)
Supplement: Supplementary file 1 [file qxag213_supplementary_data.zip › Appendix.pdf]

Appendix 1. Cumulative 12-Month Rolling Estimated Podcast Spending by the Top 1,500 Advertisers as of July 2026

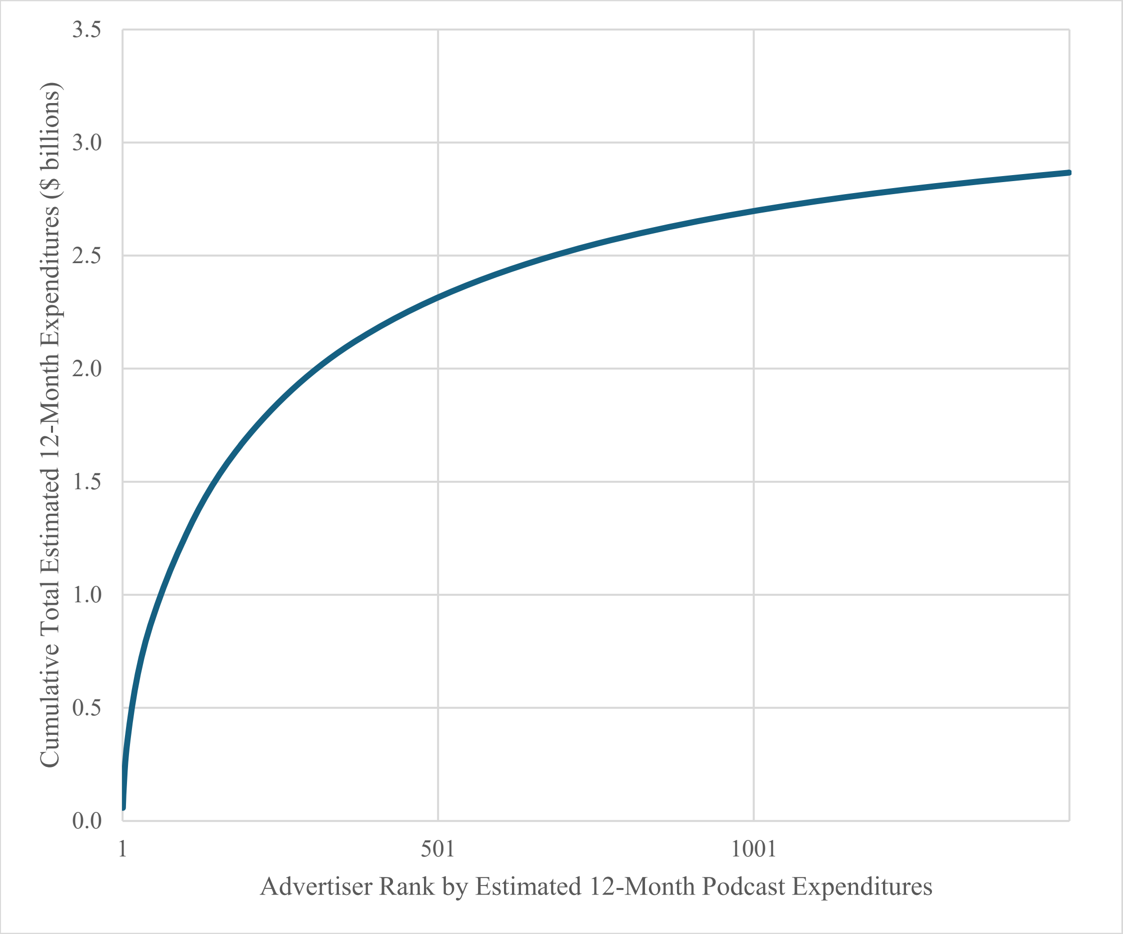

Source: Authors' analysis of Podscribe advertising occurrence data, July 2026.

Notes: Estimated spending is based on rolling 12-month estimates for the top 1,500 podcast advertisers as of July 29th, 2026.

Appendix 2. Raw Sports Gambling Advertisers, Consolidated Advertisers, and Inclusion/Exclusion Decisions

Advertiser	Consolidated To	Excluded	Exclusion Reason
Be MGM	BetMGM	Included	
Be River Sports Book	BetRivers	Included	
Be Rivers Sportsbook	BetRivers	Included	
Bet365		Included	
BetDSI Sportsbook		Included	
BetMGM		Included	
BetUS		Included	
Betano		Included	
Betfred		Included	
Betr		Included	
Betrivers	BetRivers	Included	
BettingPros		Included	
Betway		Included	
Bodog		Included	
Boyle Sports		Included	
Caliente.mx		Included	
Circa Sports		Included	
Circa Sportsbook	Circa Sports	Included	
Circa Sportsbook and Casino	Circa Sports	Included	
Cool Bet		Included	
DK Horse Racing & Betting	DraftKings	Included	
DraftKings		Included	
FanDuel		Included	
Fanatic Sportsbook	Fanatics sports book	Included	
Fanatics sports book		Included	
First Bet		Included	
Hall of Fame Bets		Included	
Ladbrokes		Included	
Life score BET squads	LiveScore Bet	Included	
LiveScore Bet		Included	
MyBookie		Included	
MyBookie AG	MyBookie	Included	
Neds		Included	
Nova Bet	Novi Bet	Included	
Novi Bet		Included	
Planetwin365		Included	
Skybet		Included	
Sports Interaction		Included	
Stake Super		Included	
Svenska Spel		Included	
Unibet		Included	
VSiN Pro		Included	
William Hill Sports Book		Included	
Winamax		Included	
XBet		Included	
bwin		Included	
1-800-Gambler		Excluded	Not a sportsbook
Action Network		Excluded	Sports betting media

Bareback Sportsbook		Excluded	Not a sportsbook
Bet Offline		Excluded	Advertiser not identifiable
BetQL		Excluded	Sports betting media
Bybetonline.Ag		Excluded	Advertiser not identifiable
Caesar's Entertainment		Excluded	Total spend <$5
DraftKings Pick6		Excluded	Fantasy sports
Elite Sports Betting		Excluded	Sports betting media
FOX Bet Super 6		Excluded	Fantasy sports
Fantasy Football today DFS podcast		Excluded	Not a sportsbook
Fox Super 6	FOX Bet Super 6	Excluded	Fantasy sports
Ftn Fantasy		Excluded	Fantasy sports
Gametime		Excluded	Not a sportsbook
Grid Rival		Excluded	Fantasy sports
Hockey Gambling Podcast		Excluded	Podcast
Oddsjam		Excluded	Sports betting media
Paramountsports.Com		Excluded	Not a sportsbook
Predicitgo		Excluded	Not a sportsbook
Prime Picks	PrizePicks	Excluded	Fantasy sports
PrizePicks		Excluded	Fantasy sports
Rake On		Excluded	Advertiser not identifiable
Sgpn		Excluded	Podcast
Sorare		Excluded	Fantasy sports
Splash Sports		Excluded	Fantasy sports
The Early Edge		Excluded	Podcast
UFL Gambling Podcast		Excluded	Podcast
Underdog Fantasy		Excluded	Fantasy sports
Unspecified Sportsbook		Excluded	Advertiser not identifiable
You Better, You Bet		Excluded	Podcast
Zip Picks		Excluded	Advertiser not identifiable

Appendix 3. Raw Alcohol Advertisers, Consolidated Advertisers, and Inclusion/Exclusion Decisions

Advertiser	Consolidated To	Excluded	Exclusion Reason
ReserveBar		Included	
1800 Tequila	Becle	Included	
Angel's Envy Bourbons		Included	
Angry Orchard	The Boston Beer Company	Included	
Anheuser Busch	Anheuser-Busch	Included	
Aspel Cider	Molson Coors	Included	
Back 40 Brewery		Included	
BeatBox Beverages	Anheuser-Busch	Included	
Beavertown Brewery	Heineken	Included	
Beer52		Included	
Beerwulf	Heineken	Included	
BlackStack Brewing		Included	
Blossa		Included	
Breck Brew		Included	
Budweiser	Anheuser-Busch	Included	
Buffalo Trace Distillery	Sazerac Company	Included	
Busch Light	Anheuser-Busch	Included	
Captain Morgan	Diageo	Included	
Carling	Molson Coors	Included	
Carlton Dry		Included	
Casamigos Tequila	Diageo	Included	
Caskers		Included	
Cayman Jack	Mark Anthony Group	Included	
Cervezas Alhambra		Included	
Copenhagen Distillery		Included	
Corona	Anheuser-Busch	Included	
Corona Extra	Anheuser-Busch	Included	
Corona Premiere	Anheuser-Busch	Included	
Country Boy Brewing		Included	
Cuervo	Becle	Included	
Daou Vineyards		Included	
Diageo Beer Company USA	Diageo	Included	
Don Julio Tequila	Diageo	Included	
Dr. Buoy Scotch Whiskey	William Grant and Sons	Included	
Dry Farm Wines		Included	
Famous Grouse	William Grant and Sons	Included	
Fireball	Sazerac Company	Included	
Fireball Whiskey	Sazerac Company	Included	
Flying Ace Spirits	CraftCo	Included	
Four Peaks Brewing Co.	Anheuser-Busch	Included	
Four Peaks Brewing Company	Anheuser-Busch	Included	
Guinness	Diageo	Included	
Guinness Zero	Diageo	Included	
HAPPY DAD		Included	
Hard AF Seltzer		Included	
Heineken		Included	
Hendricks	William Grant and Sons	Included	
Hendrick's Gin	William Grant and Sons	Included	
Hennessy		Included	

High Noon Spirits	E and J Gallo Winery	Included	
High Seltzer		Included	
Hypnotic & Anjo Tequila		Included	
Jack Daniels		Included	
Jeter Casamigos		Included	
Jim Beam	Suntory	Included	
Johnnie Walker	Diageo	Included	
Kraft Co Flying Ace Whiskey	CraftCo	Included	
Labat Blue Light	Anheuser-Busch	Included	
Labatt Blue	Anheuser-Busch	Included	
Labatt Blue Light	Anheuser-Busch	Included	
Labatt Breweries of Canada	Anheuser-Busch	Included	
Lunazul Tequila		Included	
Macadoodles		Included	
Maker's Mark	Suntory	Included	
Martel Blue Swift	Pernod Ricard	Included	
Marzadro		Included	
Michelob ULTRA	Anheuser-Busch	Included	
Mike's Hard Lemonade Company	Mark Anthony Group	Included	
Molson Coors		Included	
Noble Oak		Included	
Pabst Blue Ribbon		Included	
Padres Brewery		Included	
Pendleton Whisky	Becle	Included	
Peroni Nastro Azzurro	Asahi Group	Included	
REAL AMERICAN BEER		Included	
Remymartin		Included	
Rook and Miller Light	Molson Coors	Included	
Rossi d'Asiago		Included	
RumChata	E and J Gallo Winery	Included	
Screwball Spirits Llc	Pernod Ricard	Included	
Spritz Society		Included	
Stella Rosa Wines		Included	
Tequila Don Julio	Diageo	Included	
Tequila Don Julio 70	Diageo	Included	
The Long Drink		Included	
Tin Whiskers Beer		Included	
Tito's Handmade Vodka		Included	
Total Wine		Included	
Truly	The Boston Beer Company	Included	
Truly Hard Seltzer	The Boston Beer Company	Included	
Twisted Tea	The Boston Beer Company	Included	
Twisted Tea Brewing Company	The Boston Beer Company	Included	
Wheatley Vodka	Sazerac Company	Included	
White Claw	Mark Anthony Group	Included	
Wild Turkey		Included	
Winc		Included	
Yuengling		Included	
Bartesian		Excluded	Product not alcohol
Bottle Shop and Tasting Room		Excluded	Product not alcohol
Bud Light Morning Rush Podcast		Excluded	Podcast
Connecteam		Excluded	Product not alcohol
NUTRL		Excluded	Product not alcohol

Lulu's	Excluded	Advertiser not identifiable
Pub Pass	Excluded	Product not alcohol
Sierra Whiskey Co. with Undertack	Excluded	Product not alcohol
Spirits	Excluded	Advertiser not identifiable

Appendix 4. Monthly Podcast Episode Downloads with Advertising by Online Sportsbooks and Alcohol Advertisers, January 2024–December 2025

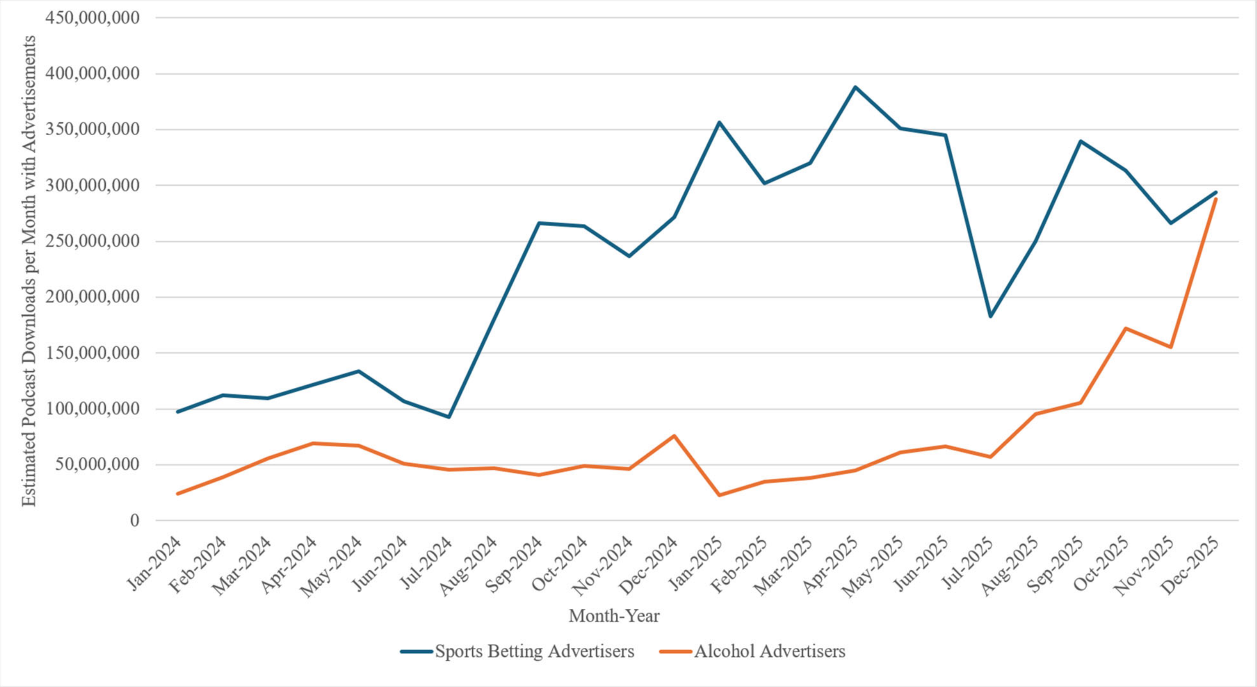

Source: Authors' analysis of Podscribe advertising occurrence data, January 2024–December 2025.

Notes: Monthly podcast episode downloads were calculated for podcasts featuring advertisements from online sportsbook advertisers and alcohol advertisers.

Appendix 5. Total Estimated Spending by Show Genre for Sports Betting and Alcohol Advertisers, 2025

Sports Betting Advertisers		Alcohol Advertisers	
**Genre**	**Spend**	**Genre**	**Spend**
Sports	$37,700,457	Sports	$13,174,404
Comedy	$23,890,762	Comedy	$6,037,658
News	$10,288,730	Culture	$4,096,268
Culture	$9,921,943	News	$1,852,579
Politics	$4,252,816	Self-Improvement	$540,437
Other	$1,982,821	Politics	$513,575
True Crime	$1,298,651	Other	$506,478
Self-Improvement	$1,167,814	True Crime	$488,455
Business	$1,055,264	Business	$426,999
Leisure	$381,406	Education	$351,154
Science	$359,707	Leisure	$252,627
Health	$341,421	Science	$154,683
Education	$257,864	Health	$145,679
Tech	$128,201	Tech	$46,474
Family	$103,258	Religion	$10,247
Religion	$58,285	Family	$4,430

Source: Authors' analysis of Podscribe advertising occurrence data, 2025.

Notes: Show genres are consolidated from Podscribe genre groupings. A single show can have multiple genres such that totals add to a greater sum than total advertising spending across industries.

Appendix 6. Demographic Composition of Podcast Audiences Exposed to Sports Betting and Alcohol Advertising, 2025

Advertiser	Male	Age	Married	Parent	White	Black	Asian	Hispanic
Sports Betting Overall	79.4%	36.2	71.9%	17.9%	88.9%	4.5%	2.6%	6.1%
Welch T-Test p-value	0.13	0.00	0.00	0.18	0.27	0.39	0.40	0.45
DraftKings	81.9%	35.4	66.8%	16.9%	88.1%	4.8%	1.5%	6.5%
FanDuel	77.3%	37.8	81.0%	20.0%	90.2%	3.8%	2.0%	5.5%
BetMGM	66.5%	35.4	74.3%	17.1%	89.7%	3.4%	1.6%	6.3%
Bet365	87.7%	36.7	75.2%	19.8%	90.4%	4.2%	1.7%	5.0%
MyBookie	88.3%	37.9	82.7%	22.0%	91.7%	4.6%	0.1%	3.4%
Ladbrokes	73.6%	35.2	69.4%	16.2%	89.2%	3.5%	2.9%	6.7%
Neds	90.2%	35.5	64.2%	13.2%	90.9%	2.4%	7.6%	3.0%
Skybet	93.7%	34.8	67.8%	20.3%	92.4%	4.3%	0.8%	3.8%
Fanatics sports book	61.6%	39.7	84.8%	20.2%	91.1%	3.1%	1.8%	5.1%
Winamax	87.0%	32.1	54.1%	2.7%	72.4%	15.0%	8.5%	9.5%
Alcohol Overall	78.6%	35.7	70.3%	17.7%	88.6%	4.7%	2.2%	6.2%
The Boston Beer Company	86.0%	34.0	55.0%	14.2%	93.6%	2.5%	1.1%	3.4%
Molson Coors	84.1%	37.0	76.7%	20.9%	87.8%	4.5%	1.7%	7.1%
Anheuser-Busch	84.1%	36.7	75.4%	16.0%	88.3%	4.7%	2.4%	6.2%
Becle	91.6%	37.6	79.0%	21.7%	86.6%	5.6%	14.5%	7.1%
HAPPY DAD	84.1%	36.0	68.4%	15.0%	87.7%	3.0%		8.4%
Jack Daniels	62.2%	35.6	75.5%	18.7%	85.3%	6.5%	2.6%	8.2%
E and J Gallo Winery	65.6%	35.6	77.2%	18.2%	86.9%	5.0%	2.7%	7.6%
Mark Anthony Group	53.2%	33.8	80.4%	14.6%	83.5%	9.0%	2.0%	7.9%
Diageo	60.5%	34.6	79.4%	17.9%	89.5%	3.5%	2.5%	5.9%
Heineken	64.4%	33.2	65.0%	16.9%	88.9%	3.6%	2.0%	7.3%

Source: Authors' analysis of Podscribe advertising occurrence data, 2025.

Notes: Demographic characteristics of podcast listening audiences were aggregated across all episodes carrying advertising from each industry in 2025, weighted by spending on episodes.

Podscribe reports only the 3 largest audience ethnicities, this leads to average listening rates per industry/advertiser by ethnicity that sum to more than 100%. P-values are from Welch's t-test comparing the two industries.
